# Healthcare resource utilization and cost of invasive pneumococcal disease, pneumonia, and acute otitis media in commercially and medicaid-insured children by risk group in the United States, 2018 to 2023

**DOI:** 10.1186/s41479-026-00206-1

**Published:** 2026-09-05

**Authors:** Salini Mohanty, Nicolae Done, Yan Song, Travis Wang, Abigail Zion, Emily Reichert, Tayler Li, Meghan White, Kelsie Cassell, Yi-Ling Huang, Kelly D. Johnson, Natalie Banniettis, Jessica P. Weaver, Kristen A. Feemster

**Affiliations:** 1 Merck & Co., Inc., 126 East Lincoln Ave, P.O. Box 2000, Rahway, NJ 07065 USA; 2 Analysis Group, Inc., Boston, MA USA

**Keywords:** Pneumococcal disease, *Streptococcus pneumoniae*, Invasive pneumococcal disease, All-cause pneumonia, Acute otitis media, Healthcare resource utilization, Costs, Healthcare administrative claims, Pediatric, Pneumococcal conjugate vaccine

## Abstract

**Background:**

Pneumococcal disease (PD) is a leading cause of morbidity among US children, particularly those with chronic medical conditions (CMC) and immunocompromising conditions (IC), and the decline in PD incidence was less pronounced in the late pneumococcal conjugate vaccine (PCV) 13 period. This study quantified healthcare resource utilization (HCRU) and costs among commercially and Medicaid-insured children with PD.

**Methods:**

This retrospective observational cohort study used Merative™ MarketScan® Commercial Database and Multi-State Medicaid Databases to identify children with invasive pneumococcal disease (IPD), all-cause pneumonia (ACP), and acute otitis media (AOM) episodes using inpatient and outpatient claims. HCRU and costs per PD episode were assessed in the commercially and Medicaid-insured populations, stratified by age (<2, 2-4, and 5-17 years) and risk group (CMC, IC, and no CMC/IC).

**Results:**

Between 2018 and 2023, commercially insured children contributed 23.6 million person-years (PY) and Medicaid-insured children contributed 23.4 million PY; 423 and 664 children had IPD, 309,540 and 281,569 had ACP, and 2,721,354 and 2,596,201 had AOM in the commercial and Medicaid populations, respectively. In both databases, HCRU and cost was higher in children with CMC or IC, compared to no CMC/IC across age groups for all PD manifestations. For IPD and ACP, inpatient admissions were 1.4 to 2.2-fold longer for children with CMC or IC. For IPD, costs per episode were 2-to-3-fold higher in children with CMC or IC, compared with those without CMC/IC (Commercial: $76,278 and $72,130 vs. $22,371; Medicaid: $17,389 and $19,945 vs. $6,476, respectively). For ACP, costs were highest in children with IC, followed by CMC and no CMC/IC (Commercial: $7,343, $2,637, and $809; Medicaid: $3,257, $1,160, and $356, respectively). AOM showed a similar pattern, with highest costs in children with IC (Commercial: $608; Medicaid: $178), followed by CMC ($442; $141), and lowest in those without CMC/IC ($368; $123).

**Conclusions:**

US children with underlying conditions that increase susceptibility to PD experience substantially higher HCRU and costs compared with those without such conditions, across all PD manifestations, regardless of insurance type and age group. These findings underscore the need for risk-based prevention strategies, including vaccination, beyond early childhood in high-risk pediatric populations.

**Supplementary Information:**

The online version contains supplementary material available at https://doi.org/10.1186/s41479-026-00206-1.

## Background

*Streptococcus pneumoniae* (*S. pneumoniae* or pneumococcus) remains a major public health concern in children, contributing substantially to morbidity, mortality, and healthcare resource utilization (HCRU) [1, 2]. Clinical manifestations of pneumococcal disease (PD) range from common localized infections, such as acute otitis media (AOM), to more serious illnesses, including pneumonia and invasive pneumococcal disease (IPD), which is defined by the isolation of *S. pneumoniae* from a normally sterile site, such as cerebrospinal fluid or blood. Since 2000, several pneumococcal conjugate vaccines (PCVs) have been licensed for prevention of PD in infants and children. The United States (US) Centers for Disease Control and Prevention’s (CDC) Advisory Committee on Immunization Practices (ACIP) recommends pneumococcal vaccination for infants and children in a standard 4-dose series administered at 2 months, 4 months, 6 months and 12 to 15 months of age; the guidelines also provide risk-based pneumococcal vaccine recommendations for children with risk conditions, including chronic medical conditions (CMC) and immunocompromising conditions (IC) [3–7]. 

Although pediatric PD incidence decreased substantially with the widespread implementation of PCVs over the past two decades, the late PCV13 period (2014–2019) was characterized by a slower decline and residual disease burden, which may be attributable to serotype replacement and breakthrough infections caused by vaccine-type serotypes [8–14]. As a result, there remains considerable HCRU and healthcare costs associated with PD among children in the US [15–17] Hu et al. estimated national annual costs of $4.3 billion for IPD, $3.6 billion for all-cause pneumonia (ACP), and $98 million for AOM, using commercial and Medicaid claims data from 2014 to 2018 [15]. 

Children with CMCs or ICs are known to experience a higher incidence of PD, compared to children with no CMC/IC,^9^ and available evidence suggest that they face greater burden of HCRU and costs associated with these PD episodes [15–24]. Existing analyses based on administrative claims or hospital data have generally reported higher utilization and costs associated with PD among children with underlying risk conditions or comorbidities, although these findings have often been constrained by limited populations, older data periods, small sample size for specific PD manifestations, or a focus on HCRU without corresponding costs evaluations [16, 17, 20, 21, 23, 25]. Moreover, evidence suggested that the prevalence of risk conditions among patients with IPD has increased over time, potentially indicating a change in the relative distribution of IPD cases between children with and without risk conditions following the broad implementation of vaccination [8, 20–22]. 

Understanding the burden associated with IPD, ACP and AOM across age and risk groups in the post-PCV13 period (2014 onward, including the COVID-19 pandemic) is essential to inform prevention strategies. To address this evidence gap, this study aimed to quantify inpatient and outpatient HCRU and costs associated with IPD, ACP, and AOM by risk group in commercially insured and Medicaid insured children, from 2018 to 2023.

## Methods

### Study design, data source and patient population

This was a retrospective observational study that evaluated HCRU and costs associated with IPD, ACP, and AOM episodes among children aged < 18 years with full-year continuous health plan enrollment during any calendar year from January 1, 2018 to December 31, 2023. Data were extracted from the Merative™ MarketScan^®^ Commercial Database and Multi-State Medicaid Databases [26]. Both databases contain information on enrollment eligibility, demographic characteristics, and medical, surgical and prescription drug utilization and expenditure data for inpatient (IP), outpatient (OP), and long-term care services. The Commercial Database captures data for individuals covered by employer-sponsored private health insurance. Approximately 350 payers across the US are represented [27]. The Multi-State Medicaid Database comprises data for Medicaid enrollees from seven to 12 geographically diverse states [27]. States included in the Medicaid Database are not publicly available, and detailed information on eligibility and population served are not disclosed due to privacy agreements, but at least one state is included from each of the four US Census regions. Both databases were included to provide more generalizable estimates across the US population. The commercial database includes employed individuals of higher income status than the Medicaid database, which includes individuals eligible through low income, disability, family-based eligibility categories, or other state-specific assistance programs.

IPD, non-invasive ACP, and AOM episodes were identified annually using IP and OP claims with ICD-10-CM codes, using the same methodology as described in previous publications [8, 10, 11, 15]. As a subset of ACP, pneumococcal pneumonia (PP) episodes were also identified; however, the incidence of PP is likely underestimated based on diagnosis codes, as pathogen-specific information is not available in claims data. For ACP and IPD, a ≥ 90-day gap with no claims using ACP or IPD-related diagnoses, respectively, was required to define the start of a new disease episode; for AOM, a ≥ 14-day gap with no claims using AOM-related diagnoses was used. As a sensitivity analysis, otitis media (OM) episodes combining AOM and otitis media with effusion (OME) diagnoses (including nonsuppurative OM, eustachian tube disorders, and myringitis) were identified using a ≥ 28-day gap to define new episodes.

IPD episodes were assessed overall and stratified by individual manifestations: meningitis, bacteremia, bacteremic pneumonia, and other IPDs (such as arthritis, osteomyelitis, peritonitis, pericarditis and endocarditis). Non-invasive ACP was assessed overall and stratified by IP and OP status. AOM and OM episodes were further categorized as non-recurrent or recurrent based on prior history of AOM or OM. Recurrent episodes were defined as ≥ 2 episodes within preceding 6 months or ≥ 3 within the preceding 12 months, with ≥ 1 episode occurring in the past 6 months [28, 29]. See Supplemental Table 1 for the specific codes used to identify PD episodes.

If there was more than one diagnosis code included on the same claim that fulfilled the definitions for more than one PD-related condition, the higher-priority condition took precedence according to the following hierarchy, to avoid double-counting. IPD episodes received the highest priority, with meningitis first, followed by bacteremia, bacteremic pneumonia, and finally other IPD. ACP was prioritized next, and AOM received the lowest priority.

### Sensitivity analysis

A sensitivity analysis was conducted among IPD episodes that required an IP stay (defined as inpatient IPD), to assess the robustness of the findings of the main analysis [28, 29]. The rationale for this approach is that the claims-based algorithm for constructing IP episodes could have captured only follow-up encounters rather than the initial hospitalization in which the IPD was initially diagnosed. The sensitivity analysis included the same outcomes as the main analysis.

### Risk groups

Children were categorized into one of three mutually exclusive risk groups within each calendar year of analysis, adapted from the CDC ACIP risk-based recommendations for pneumococcal vaccination in children: IC +/- CMC (IC with or without CMC), CMC (no IC), and no CMC/IC [7]. ICs include congenital or acquired asplenia or splenic dysfunction, congenital or acquired immunodeficiency, diseases or conditions treated with immunosuppressive drugs or radiation therapy, HIV infection, and sickle cell disease and other hemoglobinopathies. CMC includes chronic heart, liver, kidney or lung disease (including asthma of any severity), diabetes, a cerebrospinal fluid leak, or a cochlear implant.

All available data since birth (or data start: 1998 for commercial and 2001 for Medicaid, respectively) were used to identify the earliest diagnosis of risk conditions, which were carried forward across calendar years to assign mutually exclusive annual risk groups. See Supplemental Table 2 for the specific codes used to identify each risk condition.

### Study outcomes

Study outcome measures included the number of children with IPD, ACP and AOM episodes, stratified by insurance type. HCRU was reported for each PD episode, including the proportion of individuals with IP admissions, emergency room (ER) visits, OP visits, OP antibiotic prescriptions, and surgical procedures (for AOM only). In addition, the number of IP admissions per episode and mean number of IP days per admission, mean number of ER and OP visits, mean number of OP prescriptions, and mean number of surgical procedures (for AOM) were calculated for each illness episode. All HCRU results were stratified by insurance status and risk group. HCRU results were also evaluated among IPD episodes that required an IP admission.

Healthcare costs per PD episode were assessed, including the total cost, and the costs for IP admission, ER and OP visits, OP pharmacy costs, and AOM-related surgical procedures, per episode of illness. These costs were stratified by insurance status and risk group. Costs were also reported among IPD episodes that required an IP admission.

### Statistical analysis

Patients’ sociodemographic characteristics (age, sex, health plan type) and risk conditions were summarized using descriptive statistics among children with IPD, ACP, or AOM episodes, respectively, stratified by insurance type; region and urbanicity were reported for the commercially-insured population, and race/ethnicity was reported for the Medicaid-insured population.

HCRU and mean costs were summarized by PD episode type for each calendar year from 2018 to 2023 and aggregated across the entire study period. The cost per IP admission, ER visits, OP visits, OP pharmacy prescriptions, and AOM-related surgical procedures were calculated among patients who received each service. For IPD, HCRU and cost were described overall, and further stratified for each manifestation. For AOM, HCRU and cost were described overall, and further stratified between non-recurrent and recurrent AOM episodes. All results were stratified by age group (< 2, 2 to 4, 5 to 17 years) and risk group for commercially and Medicaid insured children.

Costs reflect the total paid amounts for adjudicated claims, including payer and patient out-of-pocket payments (deductibles, co-payments, and co-insurance). Costs were inflated to 2024 US dollars (USD) using the Consumer Price Index (CPI) for medical care. Statistical analyses were conducted using SAS version 9.4 (SAS Institute, Inc., Cary, North Carolina).

## Results

### Baseline characteristics

Between 2018 and 2023, an average of 4.18 million commercially insured children contributed 3.94 million PY, and an average of 4.10 million Medicaid insured children contributed 3.91 million PY at risk each year. Among the commercially insured population, 423 children experienced ≥ 1 IPD episode, 309,540 had ≥ 1 ACP episode, and 2,721,354 had ≥ 1 AOM episode. Among the Medicaid insured population, 664 children experienced ≥ 1 IPD episode, 281,569 had ≥ 1 ACP episode, and 2,596,201 had ≥ 1 AOM episode.

Sociodemographic characteristics for the commercial and Medicaid-insured populations with IPD, ACP, and AOM episodes from 2018 to 2023 are presented overall in Table 1 and stratified by risk group in Supplementary Table 3. Slightly over half of the children with PD episodes were male, regardless of insurance type. Commercially insured children with PD episodes were older (mean [SD]: 6.2 [5.1], 6.9 [4.8], 5.5 [4.6] years) than Medicaid-insured children (5.9 [5.1], 5.6 [4.5], 5.0 [4.3] years). Among children with PD episodes, those with CMC or IC were generally older than those without CMC/IC, across insurance types.

**Table 1:** Sociodemographic characteristics and risk conditions among commercially and medicaid-insured children with IPD, non-invasive ACP, and AOM episodes (2018–2023)^1^

	Commercially Insured Population			Medicaid Insured Population			
	IPD	ACP	AOM	IPD	ACP	AOM	
**Total number of patients**,** N**	423	309,540	2,721,354	664	281,569	2,596,201	
**Total number of person-years**,** N**	406	304,250	2,665,956	631	276,072	2,556,766	
**Age** ^**2**^, **mean (SD)**	6.2 (5.1)	6.9 (4.8)	5.5 (4.6)	5.9 (5.1)	5.6 (4.5)	5.0 (4.3)	
< 2 years, n (%)	112 (26.5%)	46,830 (15.1%)	752,090 (27.6%)	202 (30.4%)	70,049 (24.9%)	818,106 (31.5%)	
2–4 years, n (%)	96 (22.7%)	88,369 (28.5%)	792,122 (29.1%)	147 (22.1%)	85,106 (30.2%)	761,487 (29.3%)	
5–17 years, n (%)	215 (50.8%)	174,341 (56.3%)	1,177,142 (43.3%)	315 (47.4%)	126,414 (44.9%)	1,016,608 (39.2%)	
**Male**,** n (%)**	235 (55.6%)	162,566 (52.5%)	1,413,208 (51.9%)	395 (59.5%)	149,085 (52.9%)	1,342,601 (51.7%)	
**Health plan type**,** n (%)**							
FFS	7 (1.7%)	4,366 (1.4%)	39,845 (1.5%)	260 (39.2%)	86,269 (30.6%)	653,037 (25.2%)	
Restrictive (EPO/HMO)	59 (13.9%)	41,537 (13.4%)	335,405 (12.3%)	402 (60.5%)	193,208 (68.6%)	1,925,004 (74.1%)	
Broad (PPO/POS)	238 (56.3%)	175,425 (56.7%)	1,538,299 (56.5%)	1 (0.2%)	1,016 (0.4%)	11,044 (0.4%)	
Consumer-driven (CDHP/HDHP)	109 (25.8%)	82,309 (26.6%)	756,119 (27.8%)	-	-	-	
Missing/unknown	10 (2.4%)	5,903 (1.9%)	51,686 (1.9%)	1 (0.2%)	1,076 (0.4%)	7,116 (0.3%)	
**Region**,** n (%)**							
Northeast	64 (15.1%)	63,446 (20.5%)	417,286 (15.3%)	-	-	-	
North Central	93 (22.0%)	65,690 (21.2%)	625,577 (23.0%)	-	-	-	
South	206 (48.7%)	131,278 (42.4%)	1,310,709 (48.2%)	-	-	-	
West	57 (13.5%)	47,263 (15.3%)	355,930 (13.1%)	-	-	-	
Missing/unknown	3 (0.7%)	1,863 (0.6%)	11,852 (0.4%)	-	-	-	
**Urbanicity**,** n (%)**							
Urban	324 (76.6%)	238,979 (77.2%)	1,978,241 (72.7%)	-	-	-	
Rural	33 (7.8%)	27,825 (9.0%)	310,316 (11.4%)	-	-	-	
Missing/unknown	66 (15.6%)	42,736 (13.8%)	432,797 (15.9%)	-	-	-	
**Race/ethnicity**,** n (%)**							
White	-	-	-	237 (35.7%)	132,140 (46.9%)	1,361,813 (52.5%)	
Black	-	-	-	203 (30.6%)	69,048 (24.5%)	525,053 (20.2%)	
Hispanic	-	-	-	61 (9.2%)	24,092 (8.6%)	187,966 (7.2%)	
Other	-	-	-	35 (5.3%)	19,062 (6.8%)	164,228 (6.3%)	
Missing/unknown	-	-	-	128 (19.3%)	37,227 (13.2%)	357,141 (13.8%)	
**Risk conditions** ^**3**^, **n (%)**							
Chronic medical conditions (CMCs)							
Chronic lung disease	194 (45.9%)	92,588 (29.9%)	369,153 (13.6%)	402 (60.5%)	118,992 (42.3%)	585,941 (22.6%)	
Asthma	61 (14.4%)	70,312 (22.7%)	267,667 (9.8%)	189 (28.5%)	86,454 (30.7%)	405,816 (15.6%)	
Chronic heart disease	131 (31.0%)	15,050 (4.9%)	83,432 (3.1%)	272 (41.0%)	25,704 (9.1%)	129,022 (5.0%)	
Diabetes mellitus	7 (1.7%)	1,033 (0.3%)	6,001 (0.2%)	7 (1.1%)	1,476 (0.5%)	7,450 (0.3%)	
Chronic kidney disease	15 (3.5%)	1,047 (0.3%)	3,504 (0.1%)	31 (4.7%)	1,377 (0.5%)	4,022 (0.2%)	
Chronic liver disease	32 (7.6%)	1,348 (0.4%)	4,965 (0.2%)	56 (8.4%)	2,643 (0.9%)	9,485 (0.4%)	
Cochlear implant	21 (5.0%)	162 (0.1%)	1,190 (0.0%)	16 (2.4%)	238 (0.1%)	1,458 (0.1%)	
Cerebrospinal fluid leak	16 (3.8%)	151 (0.0%)	728 (0.0%)	17 (2.6%)	280 (0.1%)	1,240 (0.0%)	
Immunocompromising conditions (ICs)							
Congenital/acquired immunodeficiencies	79 (18.7%)	4,714 (1.5%)	17,310 (0.6%)	99 (14.9%)	4,488 (1.6%)	14,897 (0.6%)	
Diseases/conditions treated with immunosuppressive drugs/radiation therapy	36 (8.5%)	1,332 (0.4%)	4,704 (0.2%)	50 (7.5%)	1,493 (0.5%)	4,939 (0.2%)	
Sickle cell disease/other hemoglobinopathies	15 (3.5%)	1,537 (0.5%)	6,504 (0.2%)	70 (10.5%)	4,001 (1.4%)	18,766 (0.7%)	
Solid organ transplant	27 (6.4%)	916 (0.3%)	2,523 (0.1%)	39 (5.9%)	1,187 (0.4%)	2,646 (0.1%)	
Maintenance dialysis/with nephrotic syndrome	5 (1.2%)	231 (0.1%)	739 (0.0%)	18 (2.7%)	312 (0.1%)	937 (0.0%)	
Congenital/acquired asplenia/splenic dysfunction	23 (5.4%)	696 (0.2%)	2,790 (0.1%)	49 (7.4%)	1,459 (0.5%)	5,242 (0.2%)	
HIV infection	0 (0.0%)	21 (0.0%)	65 (0.0%)	1 (0.2%)	74 (0.0%)	290 (0.0%)	

Abbreviations: ACIP: Advisory Committee on Immunization Practices, AOM: Acute otitis media, CDC: Centers for Disease Control and Prevention, CDHP: Consumer-driven health plan, CMC: Chronic medical conditions, EPO: Exclusive provider organization, ER: Emergency room, FFS: Fee-for-service, HDHP: High deductible health plan, HIV: Human immunodeficiency virus, HMO: Health maintenance organization, IC: Immunocompromising conditions, IPD: Invasive pneumococcal disease, PD: Pneumococcal disease, POS: Point of service, PPO: Preferred provider organization, SD: Standard deviation

Notes:

^1^ Sociodemographic characteristics were determined at the time of the index (first-observed) episode for each calendar year, by PD manifestation

^2^ For children eligible in the year of birth, date of birth was imputed to the 15th of the first month of eligibility. For children not eligible in the year of birth, date of birth was imputed to July 1st of the year of birth

^3^ Risk conditions were adapted from the 2023 CDC’s ACIP recommendation for pneumococcal vaccination in children

### Risk conditions

Overall, the prevalence of risk conditions in children with PD manifestations was higher in Medicaid insured children, compared with commercially insured children, and those with IPD episodes were more likely to have CMC or IC compared to those with ACP or AOM episodes (Supplementary Table 3). Among children with CMC (no IC), chronic lung disease (including asthma) was the most common risk condition, occurring in at least 80%, regardless of PD manifestation or insurance type. Among children with IC +/- CMC, the most common IC risk condition was congenital or acquired immunodeficiencies.

### HCRU and costs by risk groups

#### Invasive pneumococcal disease

The HCRU and costs associated with IPD in the commercially and Medicaid insured populations from 2018 to 2023 are shown in Table 2, stratified by risk group.

**Table 2:** HCRU and costs associated with overall and inpatient IPD by risk group among commercially and Medicaid-insured children aged < 18 years (2018–2023)^1^

	Commercially Insured Population			Medicaid Insured Population		
	No CMC/IC	CMC (no IC)	IC +/- CMC	No CMC/IC	CMC (no IC)	IC +/- CMC
**Total number of IPD episodes**,** N**	***N*** **= 167**	***N*** **= 189**	***N*** **= 116**	***N*** **= 191**	***N*** **= 339**	***N*** ** = 193**
** IP admission** ^**2**^						
Episodes with at least 1 IP admission, n (%)	100 (59.9%)	129 (68.3%)	69 (59.5%)	106 (55.5%)	233 (68.7%)	133 (68.9%)
Number of IP admissions, mean (SD)	1.0 (0.0)	1.0 (0.2)	1.0 (0.2)	1.0 (0.0)	1.0 (0.1)	1.0 (0.2)
Total LOS, mean (SD)	6.2 (4.8)	12.7 (9.2)	11.9 (13.9)	6.0 (3.6)	13.4 (12.4)	12.5 (14.2)
** ER visit** ^**2**^						
Episodes with at least 1 ER visit, n (%)	103 (61.7%)	133 (70.4%)	63 (54.3%)	112 (58.6%)	231 (68.1%)	123 (63.7%)
Number of ER visits, mean (SD)	1.1 (0.3)	1.1 (0.3)	1.1 (0.3)	1.1 (0.2)	1.0 (0.2)	1.0 (0.3)
** OP visit** ^**2**^						
Episodes with at least 1 OP visit, n (%)	103 (61.7%)	89 (47.1%)	74 (63.8%)	105 (55.0%)	138 (40.7%)	92 (47.7%)
Number of OP visits, mean (SD)	3.1 (10.8)	7.5 (38.9)	2.9 (4.7)	7.4 (44.9)	4.0 (13.0)	3.6 (11.7)
** OP antibiotic prescriptions** ^**2**^						
Episodes with at least 1 prescription, n (%)	43 (25.7%)	35 (18.5%)	23 (19.8%)	45 (23.6%)	52 (15.3%)	42 (21.8%)
Number of antibiotic prescriptions, mean (SD)	1.3 (0.6)	1.4 (0.9)	1.1 (0.3)	2.0 (2.0)	1.8 (1.5)	2.4 (2.5)
**Healthcare cost (2024 USD)** ^**3**^, **per episode**						
Total costs, mean (SD)	$22,371 ($29,222)	$76,278 ($100,615)	$72,130 ($201,856)	$6,476 ($12,526)	$17,389 ($39,134)	$19,945 ($42,403)
IP admission costs, mean (SD)	$32,296 ($30,229)	$103,771 ($105,102)	$115,069 ($251,928)	$10,049 ($12,284)	$24,319 ($45,026)	$27,777 ($48,417)
ER visit costs, mean (SD)	$4,060 ($7,385)	$4,170 ($7,278)	$4,033 ($10,176)	$360 ($715)	$553 ($1,425)	$413 ($752)
OP visit costs, mean (SD)	$842 ($2,554)	$5,327 ($22,750)	$2,318 ($4,241)	$1,222 ($9,656)	$709 ($3,110)	$1,101 ($5,229)
OP pharmacy costs, mean (SD)	$34 ($70)	$40 ($58)	$74 ($137)	$77 ($197)	$60 ($91)	$72 ($113)
**Total number of inpatient IPD episodes**,** N**	*N* = 100	*N* = 129	*N* = 69	*N* = 106	*N* = 233	*N* = 133
** IP admission** ^**2**^						
Episodes with at least 1 IP admission, n (%)	100 (100.0%)	129 (100.0%)	69 (100.0%)	106 (100.0%)	233 (100.0%)	133 (100.0%)
Number of IP admissions, mean (SD)	1.0 (0.0)	1.0 (0.2)	1.0 (0.2)	1.0 (0.0)	1.0 (0.1)	1.0 (0.2)
Total LOS, mean (SD)	6.2 (4.8)	12.7 (9.2)	11.9 (13.9)	6.0 (3.6)	13.4 (12.4)	12.5 (14.2)
** ER visit** ^**2**^						
Episodes with at least 1 ER visit, n (%)	94 (94.0%)	123 (95.3%)	57 (82.6%)	98 (92.5%)	221 (94.8%)	120 (90.2%)
Number of ER visits, mean (SD)	1.1 (0.3)	1.0 (0.3)	1.1 (0.3)	1.1 (0.2)	1.0 (0.2)	1.1 (0.3)
** OP visit** ^**2**^						
Episodes with at least 1 OP visit, n (%)	43 (43.0%)	38 (29.5%)	32 (46.4%)	30 (28.3%)	41 (17.6%)	35 (26.3%)
Number of OP visits, mean (SD)	2.1 (1.3)	2.3 (1.6)	2.9 (2.7)	2.3 (3.1)	4.0 (4.5)	2.7 (3.4)
** OP antibiotic prescriptions** ^**2**^						
Episodes with at least 1 prescription, n (%)	22 (22.0%)	15 (11.6%)	9 (13.0%)	18 (17.0%)	20 (8.6%)	21 (15.8%)
Number of antibiotic prescriptions, mean (SD)	1.3 (0.7)	1.3 (0.6)	1.0 (0.0)	2.1 (1.9)	1.8 (1.4)	2.0 (1.3)
**Healthcare cost (2024 USD)** ^**3**^, **per episode**						
Total costs, mean (SD)	$36,288 ($30,577)	$107,990 ($106,297)	$119,583 ($251,497)	$10,453 ($12,439)	$24,940 ($45,194)	$28,333 ($48,687)
IP admission costs, mean (SD)	$32,296 ($30,229)	$103,771 ($105,102)	$115,069 ($251,928)	$10,049 ($12,284)	$24,319 ($45,026)	$27,777 ($48,417)
ER visit costs, mean (SD)	$4,009 ($7,607)	$3,758 ($4,690)	$3,989 ($10,540)	$351 ($755)	$552 ($1,436)	$407 ($758)
OP visit costs, mean (SD)	$499 ($663)	$2,144 ($4,063)	$2,602 ($4,664)	$252 ($433)	$535 ($1,040)	$680 ($2,875)
OP pharmacy costs, mean (SD)	$38 ($94)	$36 ($46)	$91 ($182)	$51 ($81)	$47 ($63)	$57 ($47)

Abbreviations: CDHP: Consumer-driven health plan, CMC: Chronic medical condition, EPO: Exclusive provider organization, ER: Emergency room, HDHP: High deductible health plan, HMO: Health maintenance organization, HCRU: Healthcare resource utilization, IC: Immunocompromising condition, IP: Inpatient, IPD: Invasive pneumococcal disease, LOS: Length of stay, OP: Outpatient, POS: Point of service, PPO: Preferred provider organization, SD: Standard deviation, USD: United States dollar

Notes:

^1^ Both capitated and non-capitated plans were included in the HCRU and cost analyses. Non-capitated plans include EPO, PPO, POS, HDHP, and CDHP. Capitated plans include HMO

^2^ The numbers of IP admission, total LOS (i.e., IP days), ER visit, OP visit, and OP antibiotic prescriptions were calculated among episodes with at least one corresponding visit

^3^ The healthcare costs included the total payments from payers and out-of-pocket payments by patients. The healthcare costs per episode for IP admission, ER visit, OP visit, and OP pharmacy were calculated among episodes with at least one corresponding visit. Costs are reported in 2024 USD

Among commercially insured children with IPD, the majority of episodes required IP admission. IP days per episode was longer in the CMC (no IC) and IC +/- CMC groups (mean: 12.7 days, 11.9 days), compared to the no CMC/IC group (mean: 6.2 days). A similar pattern was observed among Medicaid-insured children with IPD.

Total treatment costs per IPD episode were substantially higher for children in the CMC (no IC) and IC +/- CMC groups, compared to the no CMC/IC group, regardless of insurance type. Among commercially insured children, mean total treatment costs were over 3-fold higher in the CMC (no IC) and IC +/- CMC groups compared with the no CMC/IC group ($76,278 and $72,130, respectively versus $22,371). Among Medicaid-insured children, mean total costs were more than double in the CMC (no IC) and IC +/- CMC groups, compared with the no CMC/IC group ($17,389 and $19,945, respectively versus $6,476). IP admissions comprised most of the total costs in both insurance types for children in all three risk groups. The mean cost of IP hospitalization was highest in the IC +/- CMC group (Commercial: $115,069; Medicaid: $27,777), followed by the CMC (no IC) group ($103,771; $24,319), and the no CMC/IC group ($32,296; $10,049).

HCRU and costs associated with specific IPD manifestations, including meningitis, bacteremia, bacteremic pneumonia, and other IPD by risk group are presented in Supplementary Table 4. HCRU and costs associated with overall IPD and its manifestations by risk group and age group are presented in Supplementary Table 5. Among commercially insured children, total costs for IPD episodes were highest in children < 2 years of age, particularly in the CMC (no IC) and IC +/- CMC groups; in contrast, total costs for IPD episodes were higher in children aged 2–4 and 5–17 years among Medicaid-insured children.

HCRU and costs associated with overall IPD, meningitis, bacteremia, bacteremic pneumonia, and other IPD by risk group, age group, and calendar year from 2018 to 2023 are presented in Supplementary Tables 6 through 9.

### Sensitivity analysis of invasive pneumococcal disease with inpatient admission

The HCRU and costs associated with inpatient IPD in the commercially and Medicaid-insured populations from 2018 to 2023 are shown in Table 2, stratified by risk group. Among all IPD episodes occurring in commercially and Medicaid-insured children, 63% and 65% involved an IP admission, respectively. Mean total costs per IPD episode were higher for children requiring IP admission, compared with the overall cohort, across insurance types and risk groups.

As for the overall cohort, total treatment costs per inpatient IPD episode were substantially higher for children in the CMC (no IC) and IC +/- CMC groups, regardless of insurance type. Among commercially insured children, mean total costs were 3 to 4-fold higher in the CMC (no IC) and IC +/- CMC groups ($107,990 and $119,583) compared to the no CMC/IC group ($36,288). Among Medicaid-insured children, total costs were more than double in the CMC (no IC) and IC +/- CMC groups, compared with the no CMC/IC group ($24,940 and $28,333 versus $10,453).

HCRU and costs for inpatient IPD manifestations (i.e., meningitis, bacteremia, bacteremic pneumonia, and other IPD) by risk group are presented in Supplementary Table 10. HCRU and costs associated with inpatient IPD and specific manifestations (i.e., meningitis, bacteremia, bacteremic pneumonia, and other IPD) by risk group and age group are presented in Supplementary Table 11. HCRU and costs associated with inpatient IPD and specific manifestations are further stratified by risk group, age group, and year in Supplementary Tables 12–15.

### All-cause pneumonia

The HCRU and costs associated with ACP in the commercially and Medicaid-insured populations from 2018 to 2023 are shown in Table 3, stratified by risk group.

**Table 3:** HCRU and costs associated with overall ACP, inpatient ACP, and outpatient ACP by risk group among commercially and Medicaid- insured children aged < 18 years (2018–2023)^1^

	Commercially Insured Population			Medicaid Insured Population		
	No CMC/IC	CMC (no IC)	IC +/- CMC	No CMC/IC	CMC (no IC)	IC +/- CMC
**Total number of overall ACP episodes**,** N**	***N*** ** = 211,421**	***N*** **= 99,806**	***N*** **= 7,500**	***N*** **= 154,579**	***N*** **= 125**,**984**	***N*** **= 9,886**
** IP admission** ^**2**^						
Episodes with at least 1 IP admission, n (%)	3,575 (1.7%)	6,402 (6.4%)	923 (12.3%)	4,444 (2.9%)	11,255 (8.9%)	1,785 (18.1%)
Number of IP admissions, mean (SD)	1.0 (0.1)	1.0 (0.2)	1.1 (0.4)	1.0 (0.1)	1.0 (0.2)	1.1 (0.3)
Total LOS, mean (SD)	3.3 (1.9)	4.7 (5.9)	6.5 (13.4)	3.5 (1.9)	5.3 (6.6)	7.0 (11.8)
** ER visit** ^**2**^						
Episodes with at least 1 ER visit, n (%)	49,504 (23.4%)	29,315 (29.4%)	2,612 (34.8%)	78,357 (50.7%)	65,058 (51.6%)	5,367 (54.3%)
Number of ER visits, mean (SD)	1.1 (0.4)	1.2 (0.5)	1.2 (0.6)	1.1 (0.3)	1.1 (0.4)	1.2 (0.4)
** OP visit** ^**2**^						
Episodes with at least 1 OP visit, n (%)	178,150 (84.3%)	83,110 (83.3%)	5,982 (79.8%)	96,457 (62.4%)	82,110 (65.2%)	6,286 (63.6%)
Number of OP visits, mean (SD)	1.3 (0.8)	1.5 (2.3)	1.8 (3.3)	1.4 (2.0)	1.6 (5.1)	2.0 (6.5)
** OP antibiotic prescriptions** ^**2**^						
Episodes with at least 1 prescription, n (%)	168,996 (79.9%)	76,652 (76.8%)	5,122 (68.3%)	112,120 (72.5%)	88,896 (70.6%)	6,063 (61.3%)
Number of antibiotic prescriptions, mean (SD)	1.2 (0.4)	1.3 (0.6)	1.4 (0.8)	1.2 (0.4)	1.2 (0.6)	1.4 (1.4)
**Healthcare cost (2024 USD)** ^**3**^, **per episode**						
Total costs, mean (SD)	$809 ($3,309)	$2,637 ($15,703)	$7,343 ($64,638)	$356 ($1,195)	$1,160 ($7,849)	$3,257 ($26,238)
IP admission costs, mean (SD)	$13,656 ($14,604)	$25,929 ($52,846)	$47,023 ($177,114)	$4,140 ($4,678)	$9,131 ($19,670)	$15,359 ($59,905)
ER visit costs, mean (SD)	$1,557 ($2,866)	$2,136 ($4,471)	$2,647 ($6,810)	$289 ($560)	$378 ($897)	$470 ($1,140)
OP visit costs, mean (SD)	$232 ($726)	$388 ($2,098)	$764 ($4,191)	$123 ($323)	$201 ($5,342)	$304 ($1,932)
OP pharmacy costs, mean (SD)	$23 ($117)	$32 ($362)	$38 ($186)	$20 ($33)	$28 ($181)	$69 ($854)
**Total number of inpatient ACP episodes**,** N**	***N*** **= 3,575**	***N*** ** = 6,402**	***N*** **= 923**	***N*** ** = 4,444**	***N*** **= 11,255**	***N*** **= 1,785**
** IP admission** ^**2**^						
Episodes with at least 1 IP admission, n (%)	3,575 (100.0%)	6,402 (100.0%)	923 (100.0%)	4,444 (100.0%)	11,255 (100.0%)	1,785 (100.0%)
Number of IP admissions, mean (SD)	1.0 (0.1)	1.0 (0.2)	1.1 (0.4)	1.0 (0.1)	1.0 (0.2)	1.1 (0.3)
Total LOS, mean (SD)	3.3 (1.9)	4.7 (5.9)	6.5 (13.4)	3.5 (1.9)	5.3 (6.6)	7.0 (11.8)
** ER visit** ^**2**^						
Episodes with at least 1 ER visit, n (%)	3,168 (88.6%)	5,851 (91.4%)	820 (88.8%)	4,118 (92.7%)	10,352 (92.0%)	1,580 (88.5%)
Number of ER visits, mean (SD)	1.3 (0.6)	1.3 (0.6)	1.3 (0.7)	1.2 (0.5)	1.2 (0.6)	1.3 (0.6)
** OP visit** ^**2**^						
Episodes with at least 1 OP visit, n (%)	2,632 (73.6%)	4,276 (66.8%)	540 (58.5%)	2,563 (57.7%)	5,764 (51.2%)	819 (45.9%)
Number of OP visits, mean (SD)	2.0 (1.9)	2.5 (4.0)	3.2 (6.2)	1.9 (3.6)	2.5 (15.9)	3.3 (14.0)
** OP antibiotic prescriptions** ^**2**^						
Episodes with at least 1 prescription, n (%)	2,094 (58.6%)	3,250 (50.8%)	407 (44.1%)	1,871 (42.1%)	4,151 (36.9%)	586 (32.8%)
Number of antibiotic prescriptions, mean (SD)	1.6 (0.8)	1.7 (1.1)	2.0 (1.4)	1.5 (0.8)	1.7 (1.4)	2.1 (2.3)
**Healthcare cost (2024 USD)** ^**3**^, **per episode**						
Total costs, mean (SD)	$16,840 ($16,171)	$29,756 ($54,389)	$51,410 ($177,896)	$4,563 ($4,891)	$9,765 ($19,999)	$16,194 ($60,039)
IP admission costs, mean (SD)	$13,656 ($14,604)	$25,929 ($52,846)	$47,023 ($177,114)	$4,140 ($4,678)	$9,131 ($19,670)	$15,359 ($59,905)
ER visit costs, mean (SD)	$3,005 ($5,660)	$3,266 ($6,970)	$3,888 ($11,223)	$318 ($897)	$455 ($1,494)	$548 ($1,741)
OP visit costs, mean (SD)	$684 ($2,942)	$1,198 ($4,489)	$1,545 ($5,959)	$202 ($695)	$372 ($2,405)	$622 ($4,566)
OP pharmacy costs, mean (SD)	$43 ($145)	$102 ($1,547)	$65 ($153)	$29 ($65)	$71 ($587)	$218 ($2,199)
**Total number of outpatient ACP episodes**,** N**	***N*** **= 207,846**	***N*** ** = 93,404**	***N*** **= 6,577**	***N*** **= 150,135**	***N*** **= 114,729**	***N*** **= 8,101**
** IP admission** ^**2**^						
Episodes with at least 1 IP admission, n (%)	0 (0.0%)	0 (0.0%)	0 (0.0%)	0 (0.0%)	0 (0.0%)	0 (0.0%)
Number of IP admissions, mean (SD)	-	-	-	-	-	-
Total LOS, mean (SD)	-	-	-	-	-	-
** ER visit** ^**2**^						
Episodes with at least 1 ER visit, n (%)	46,336 (22.3%)	23,464 (25.1%)	1,792 (27.2%)	74,239 (49.4%)	54,706 (47.7%)	3,787 (46.7%)
Number of ER visits, mean (SD)	1.1 (0.4)	1.2 (0.5)	1.2 (0.5)	1.1 (0.3)	1.1 (0.4)	1.1 (0.3)
** OP visit** ^**2**^						
Episodes with at least 1 OP visit, n (%)	175,518 (84.4%)	78,834 (84.4%)	5,442 (82.7%)	93,894 (62.5%)	76,346 (66.5%)	5,467 (67.5%)
Number of OP visits, mean (SD)	1.3 (0.7)	1.5 (2.2)	1.7 (2.8)	1.3 (1.9)	1.5 (3.0)	1.8 (4.3)
** OP antibiotic prescriptions** ^**2**^						
Episodes with at least 1 prescription, n (%)	166,902 (80.3%)	73,402 (78.6%)	4,715 (71.7%)	110,249 (73.4%)	84,745 (73.9%)	5,477 (67.6%)
Number of antibiotic prescriptions, mean (SD)	1.2 (0.4)	1.2 (0.5)	1.3 (0.7)	1.2 (0.4)	1.2 (0.6)	1.4 (1.3)
**Healthcare cost (2024 USD)** ^**3**^, **per episode**						
Total costs, mean (SD)	$533 ($1,465)	$778 ($2,626)	$1,159 ($4,071)	$232 ($473)	$316 ($4,520)	$407 ($1,114)
IP admission costs, mean (SD)	-	-	-	-	-	-
ER visit costs, mean (SD)	$1,458 ($2,536)	$1,855 ($3,530)	$2,079 ($2,999)	$287 ($535)	$364 ($730)	$437 ($757)
OP visit costs, mean (SD)	$225 ($634)	$345 ($1,873)	$686 ($3,965)	$120 ($306)	$188 ($5,501)	$257 ($1,074)
OP pharmacy costs, mean (SD)	$23 ($116)	$28 ($141)	$36 ($189)	$20 ($32)	$25 ($112)	$48 ($382)

Abbreviations: ACP: All-cause pneumonia, CDHP: Consumer-driven health plan, CMC: Chronic medical condition, EPO: Exclusive provider organization, ER: Emergency room, HDHP: High deductible health plan, HMO: Health maintenance organization, HCRU: Healthcare resource utilization, IC: Immunocompromising condition, IP: Inpatient, LOS: Length of stay, OP: Outpatient, POS: Point of service, PPO: Preferred provider organization, SD: Standard deviation, USD: United States dollar

Notes:

^1^ Both capitated and non-capitated plans were included in the HCRU and cost analyses. Non-capitated plans include EPO, PPO, POS, HDHP, and CDHP. Capitated plans include HMO

^2^ The numbers of IP admission, total LOS (i.e., IP days), ER visit, OP visit, and OP antibiotic prescriptions were calculated among episodes with at least one corresponding visit

^3^ The healthcare costs included the total payments from payers and out-of-pocket payments by patients. The healthcare costs per episode for IP admission, ER visit, OP visit, and OP pharmacy were calculated among episodes with at least one corresponding visit. Costs are reported in 2024 USD

Among commercially insured children with ACP, IP admissions were most common in the IC +/- CMC group (12.3%), followed by the CMC (no IC) group (6.4%), and the no CMC/IC group (1.7%); the number of IP days per episode was also higher for the IC and CMC (no IC) groups (mean: 6.5 days, 4.7 days), compared to the no CMC/IC group (3.3 days). A similar pattern was observed among Medicaid-insured children with ACP. OP visits were more common in commercially insured children, and ER visits were more common in Medicaid insured children, across risk groups. OP antibiotics were prescribed in the majority of ACP episodes, regardless of insurance type (Commercial: 68%-80%, Medicaid: 61%-73%), with similar numbers of antibiotic prescriptions per episode across risk groups.

Regardless of insurance type, total cost per ACP episode was highest in the IC +/- CMC group (Commercial: $7,343; Medicaid: $3,257), followed by the CMC (no IC) group (Commercial: $2,637; Medicaid: $1,160), and the no CMC/IC group (Commercial: $809; Medicaid: $356). This trend persisted across both inpatient and outpatient ACP episodes, with substantially higher costs for the IC +/- CMC and CMC (no IC) groups, compared with the no CMC/IC group, though the average costs per outpatient ACP were relatively small.

HCRU and costs associated with overall PP, inpatient PP, and outpatient PP by risk group are presented in Supplementary Table 16. HCRU and costs associated with overall ACP, inpatient ACP, and outpatient ACP, as well as overall PP, inpatient PP, and outpatient PP, further stratified by risk group and age group are presented in Supplementary Tables 5 and further stratified by risk group, age group, and year in Supplementary Tables 6–9. However, only 0.33% (*n* = 1,037/318,727) of ACP episodes in the Commercial database and 0.47% (*n* = 1,362/290,449) in the Medicaid database were recorded as PP, reflecting the limited use of pathogen-specific coding in claims data. While the small number of PP episodes limits precision, inpatient admission rates and costs per episode for PP were generally higher than for overall ACP in the CMC and IC groups, consistent with pathogen-confirmed cases representing more severe presentations.

### Acute otitis media

The HCRU and costs associated with AOM in the commercially and Medicaid-insured populations from 2018 to 2023 are shown in Table 4, stratified by risk group. HCRU was similar across risk groups in both insurance types. Fewer than 0.1% of AOM episodes included an IP admission. There was approximately one ER or OP visit per AOM episode across risk groups. In the commercially insured population, older children had a higher proportion of ER visits but lower proportion of OP visits across risk groups; these results fluctuated among Medicaid-insured children. Most episodes included OP antibiotic prescriptions (Commercial: 73% to 77%; Medicaid: 73% to 76%), with a similar number of antibiotic prescriptions across risk groups. AOM-related surgical procedures were more frequent in the IC +/- CMC group (Commercial: 5.9%; Medicaid: 8.6%) and CMC (no IC) group (4.7%; 8.9%) compared to the no CMC/IC group (3.9%; 6.2%), with a similar number of surgical procedures across risk groups in both insurance types.

**Table 4:** HCRU and costs associated with overall AOM by risk group among commercially and medicaid-insured children aged < 18 years (2018–2023)^1^

	Commercially Insured Population			Medicaid Insured Population		
	No CMC/IC	CMC (no IC)	IC +/- CMC	No CMC/IC	CMC (no IC)	IC +/- CMC
**Total number of AOM episodes**,** N**	***N*** **= 3,385**,**240**	***N*** ** = 654,438**	***N*** **= 43,774**	***N*** **= 1,189**,**450**	***N*** **= 158,771**	***N*** **= 10,123**
** IP admission** ^**2**^						
Episodes with at least 1 IP admission, n (%)	215 (0.0%)	141 (0.0%)	37 (0.1%)	114 (0.0%)	63 (0.0%)	10 (0.1%)
Number of IP admissions, mean (SD)	1.0 (0.1)	1.0 (0.0)	1.0 (0.0)	1.0 (0.1)	1.0 (0.0)	1.0 (0.0)
Total LOS, mean (SD)	2.8 (1.6)	2.9 (1.4)	3.6 (3.7)	2.7 (1.4)	2.8 (1.1)	3.1 (1.2)
** ER visit** ^**2**^						
Episodes with at least 1 ER visit, n (%)	570,492 (16.9%)	114,360 (17.5%)	7,610 (17.4%)	148,725 (12.5%)	21,047 (13.3%)	1,541 (15.2%)
Number of ER visits, mean (SD)	1.0 (0.2)	1.0 (0.2)	1.1 (0.3)	1.1 (0.3)	1.1 (0.3)	1.1 (0.3)
** OP visit** ^**2**^						
Episodes with at least 1 OP visit, n (%)	2,873,580 (84.9%)	553,738 (84.6%)	37,180 (84.9%)	1,070,691 (90.0%)	143,046 (90.1%)	8,946 (88.4%)
Number of OP visits, mean (SD)	1.2 (0.7)	1.2 (0.9)	1.2 (1.0)	1.3 (0.7)	1.3 (0.8)	1.3 (0.9)
** OP antibiotic prescriptions** ^**2**^						
Episodes with at least 1 prescription, n (%)	2,598,493 (76.8%)	504,892 (77.1%)	32,124 (73.4%)	902,175 (75.8%)	118,184 (74.4%)	7,408 (73.2%)
Number of antibiotic prescriptions, mean (SD)	1.1 (0.3)	1.1 (0.3)	1.1 (0.4)	1.1 (0.4)	1.2 (0.4)	1.2 (0.5)
** AOM-related surgical procedures** ^**2**^						
Episodes with at least 1 AOM-related surgical procedure, n (%)	131,236 (3.9%)	30,904 (4.7%)	2,592 (5.9%)	74,074 (6.2%)	14,082 (8.9%)	867 (8.6%)
Number of AOM-related surgical procedures, mean (SD)	1.0 (0.1)	1.0 (0.1)	1.0 (0.1)	1.0 (0.1)	1.0 (0.1)	1.0 (0.1)
**Healthcare cost (2024 USD)** ^**3**^, **per episode**						
Total costs, mean (SD)	$368 ($1,158)	$442 ($1,640)	$608 ($3,441)	$123 ($304)	$141 ($687)	$178 ($713)
IP admission costs, mean (SD)	$11,639 ($14,318)	$12,485 ($10,319)	$16,840 ($28,218)	$2,988 ($3,381)	$3,984 ($13,968)	$4,490 ($5,853)
ER visit costs, mean (SD)	$381 ($780)	$448 ($956)	$568 ($1,158)	$122 ($207)	$135 ($244)	$167 ($371)
OP visit costs, mean (SD)	$213 ($624)	$247 ($940)	$314 ($1,321)	$89 ($242)	$101 ($500)	$121 ($477)
OP pharmacy costs, mean (SD)	$20 ($38)	$23 ($57)	$25 ($46)	$15 ($24)	$17 ($32)	$18 ($36)
AOM surgical procedure costs, mean (SD)	$2,767 ($3,295)	$2,851 ($4,754)	$3,560 ($11,641)	$352 ($855)	$353 ($2,212)	$431 ($2,078)

Abbreviations: AOM: Acute otitis media, CDHP: Consumer-driven health plan, CMC: Chronic medical condition, EPO: Exclusive provider organization, ER: Emergency room, HDHP: High deductible health plan, HMO: Health maintenance organization, HCRU: Healthcare resource utilization, IC: Immunocompromising condition, IP: Inpatient, LOS: Length of stay, OP: Outpatient, POS: Point of service, PPO: Preferred provider organization, SD: Standard deviation, USD: United States dollar

Notes:

^1^ Both capitated and non-capitated plans were included in the HCRU and cost analyses. Non-capitated plans include EPO, PPO, POS, HDHP, and CDHP. Capitated plans include HMO

^2^ The numbers of IP admission, total LOS (i.e., IP days), ER visit, OP visit, OP antibiotic prescriptions, and AOM-related surgical procedures were calculated among episodes with at least one corresponding visit

^3^ The healthcare costs included the total payments from payers and out-of-pocket payments by patients. The healthcare costs per episode for IP admission, ER visit, OP visit, OP pharmacy, and AOM-related surgical procedures were calculated among episodes with at least one corresponding visit. Costs are reported in 2024 USD

In commercially and Medicaid-insured children, total costs per AOM episode were highest in the IC +/- CMC group (Commercial: $608; Medicaid: $178), followed by the CMC (no IC) group ($442; $141), and the no CMC/IC group ($368; $123); the costs of AOM-related surgical procedures mirrored this trend (IC +/- CMC: $3,560; $431; CMC (no IC): $2,851; $353; no CMC/IC: $2,767; $352). Total costs and surgical costs were higher for commercially insured children across risk groups. In both insurance types, recurrent AOM episodes consistently incurred higher costs than non-recurrent episodes, with a similar stepwise cost pattern observed across risk groups for both types of episodes (Supplementary Table 17). HCRU and costs associated with overall AOM, non-recurrent AOM, and recurrent AOM stratified further by risk group and age group are presented in Supplementary Tables 5, and by risk group, age group, and year in Supplementary Tables 6 to 9. Across both insurance types, HCRU remained relatively stable over time, although fluctuations were observed between 2020 and 2021 across all risk groups.

HCRU and costs associated with overall OM, non-recurrent OM, and recurrent OM episodes, both overall and stratified by risk group, age group, and year are presented in Supplementary Tables 18–23. The patterns in HCRU and costs were similar to those observed for AOM across insurance types, age groups, and risk groups.

## Discussion

This study found that healthcare utilization and costs associated with pneumococcal disease in the US pediatric population remain high, particularly among children with CMC and IC. In both commercially and Medicaid-insured children, IPD, ACP, and AOM episodes in children with CMC and IC were associated with higher total costs, more frequent hospitalization, and longer length of stay, compared with children with no CMC/IC. Mean total costs per IPD episode were 2- to 3-fold higher for children in the CMC (no IC) and IC +/- CMC groups, compared to the no CMC/IC group, with most costs attributed to IP admissions. Total costs for ACP were also much higher (3- to 9-fold) in children with risk conditions. The same trend was observed for costs for AOM, and surgical procedures were also more common in the CMC (no IC) and IC +/- CMC groups. This pattern of higher HCRU and cost for children with CMC or IC, versus no CMC/IC persisted across all age groups for all PD manifestations, regardless of insurance type. Total costs were generally higher for children < 2 years of age, across risk groups and insurance types. Asthma constituted a substantial proportion of the CMC group across PD manifestations, but it was identified using a simplified definition that did not distinguish severity, which differs from the moderate-to-severe asthma definition specified by CDC ACIP. As a result, future research is warranted to examine PD burden by asthma severity.

Additionally, this study found that a substantial proportion of pneumococcal disease episodes occurred among children older than 2 years. This pattern was observed across all risk groups and was more pronounced among children with CMC or IC, indicating that PD risk extends beyond early childhood. This finding is consistent with a surveillance data study, which found children with comorbidities were older at the time of IPD diagnosis [30]. 

These results are consistent with the evidence that children of young age and those with CMC and IC face an increased burden and severity of PD, compared to older children, and those with no CMC/IC [18–22, 24]. Higher hospitalization costs and longer length of stay for PD among young children and those with risk conditions have also been reported in other studies [16, 17, 23]. Mohanty et al. (2024) conducted a study which utilized a database of US children hospitalized for IPD, ACP, or AOM [17]. The presence of IPD (vs. non-invasive ACP/AOM), age < 2 years, and moderate or high risk condition (similar to CMC or IC in the current study), were associated with a longer length of stay; the presence of IPD, having a moderate or having a high risk condition was associated with higher cost per admission [17]. Weycker et al. (2016) also reported higher costs for PD in children with risk conditions in a study that utilized three independent claims databases; compared with children with no risk conditions, mean costs per inpatient ACP episode were almost 2-fold higher in children with at-risk conditions, and 3-fold higher in those with high-risk conditions [23]. A study by Averin et al. (2025), which utilized Optum claims of commercially insured children, found that the total episodic cost of ACP requiring IP admission was nearly 2-fold higher in children with risk conditions vs. those with no risk conditions [16]. In a study using Massachusetts surveillance data, Iroh Tam et al. (2014) found that 20% of children with IPD in the pre-PCV13 and 24% in the post-PCV13 phase had a risk condition; compared with children with no risk conditions, they were more frequently hospitalized (80% vs. 50%) and had longer stays (median: 3 vs. 0.5 days), though the sample size was limited [25]. 

The current study reported costs per episode for inpatient IPD by risk group for Medicaid-insured children that are comparable to the adjusted mean costs per admission for IPD reported in the Mohanty et al. (2024) study, while these costs for the commercially insured children was higher in the current study. This difference may be attributable to the relatively small sample sizes in the Mohanty study, which included 4,575 patients from 57 hospitals, with 36 (0.8%) patients hospitalized with IPD, as well as the difference in study populations and data sources [17]. The Averin study reported higher mean costs for IPD episodes requiring hospitalization among children without comorbidities ($58,099) compared with the no CMC/IC group ($36,288) in the current study. In contrast, among children with comorbidities, Averin et al. reported lower costs ($43,450) than those in the CMC (no IC) ($107,990) and IC +/- CMC ($119,583) groups. However, these comparisons should be interpreted with caution given the sample size for IPD was small (25 and 68 children with and without comorbidities, respectively) [16]. 

In addition, Averin et al. reported similar inpatient ACP episode costs among children without risk conditions [16]. Among children with risk conditions, the reported episodic costs of hospitalized ACP ($28,095) were lower than the inpatient ACP episode costs observed in the CMC (no IC) group ($29,756) and IC +/- CMC group ($51,410) in the current study [16]. Even after accounting for an estimated inflation of approximately 13% from 2019 to 2024, the episodic costs remained different. These differences likely reflect methodological differences, including risk group definitions and data sources. Specifically, Averin et al. classified children as with versus without comorbidities based on the CDC-specified risk conditions and medications for PD and utilized the Optum data from 2015 to 2019, which predominantly captures UnitedHealthcare enrollees. In contrast, the current study classified patients into IC +/- CMC, CMC (no IC), and no CMC/IC groups and used MarketScan data from 2018 to 2023, encompassing multiple payers. Separately, the episode costs for hospitalized AOM reported by Averin et al. were comparable to the IP admission costs among AOM episodes in the commercially insured children.

The current study provided recent evidence on the burden of IPD, ACP, and AOM episodes over a 6-year period. HCRU and costs per PD episode varied by risk group, PD manifestation, and insurance type. Although PD prevention primarily targets children under 5, our age-stratified analyses showed that elevated HCRU and costs per episode among higher-risk children persisted across all age groups, consistent with findings reported by Averin et al. [16] This underscores the importance of risk-based pneumococcal vaccination recommendations beyond early childhood [7]. 

Strengths of this study include the use of large databases from a recent 6-year period, providing updated estimates of episode-level HCRU and costs across the post-PCV13 period and encompassing the COVID-19 pandemic years through 2023. The inclusion of both commercial and Medicaid populations captures a broad socioeconomic spectrum, enhancing the generalizability of findings to the insured US pediatric population. These results offer timely evidence on the burden of PD episodes as newer PCVs are introduced, and are important to guide prevention efforts, as PD incidence may be impacted by factors such as serotype replacement.

As with any administrative claims data analysis, this study is subject to limitations. Miscoding of diagnoses, procedures, and prescriptions may occur, potentially leading to misclassification of PD claims, surgical procedures, and antibiotic prescriptions. In addition, *S. pneumoniae*-specific IPD episodes were identified using diagnosis codes without confirmation from lab results and medical charts. Although an established algorithm was used to identify PD episodes, underlying assumptions may not fully reflect true clinical episodes, potentially leading to misclassification of episode timing and duration, which may have affected HCRU and cost estimates. In addition, some IPD episodes were not reported to include hospitalizations; due to data limitations, we were not able to identify the index hospitalization in these cases, given changes in insurance coverage over time in both the Commercial and Medicaid databases. Moreover, all states were not included in the Medicaid database, though the sample is from geographically diverse states. Furthermore, for children without continuous coverage since birth, diagnoses may be left-truncated, capturing only the first recorded claim, which may lead to potential misclassification of risk group. Additionally, to avoid double counting, disease episodes were defined using a clinical severity-based hierarchy; however, in cases with overlapping conditions, HCRU and costs may have been misattributed. Moreover, all etiologies of pneumonia and AOM were included, which may overestimate the burden attributable to *S. pneumoniae*. In addition, we classified asthma of any severity as a risk condition, rather than limiting it to moderate-to-severe asthma, as in the ACIP guidelines; because children with mild asthma may have a lower underlying PD risk than those with moderate-to-severe asthma, this approach would tend to attenuate, rather than inflate, the observed differences between risk groups. Lastly, the study findings were limited to children enrolled in commercial and Medicaid insurance in the US, and may not be generalizable to uninsured populations. In addition, individual-level pneumococcal vaccination status was not available in the claims data, as immunization records are typically maintained separately from administrative claims. We note, however, that US pediatric PCV coverage has been high during the study period [31]. 

## Conclusions

This study provides robust, recent evidence that US children in all age groups with chronic medical or immunocompromising conditions face a disproportionate burden of pneumococcal disease, both clinically and economically. The burden is reflected in higher hospitalization rates, more IP days per admission, and higher treatment costs across IPD, ACP, and AOM episodes, compared to children without CMC/IC. These findings highlight the need for targeted prevention and enhanced care strategies for children with risk conditions beyond the early childhood years to help reduce hospitalizations, costs, and long-term impacts on patients, families and the healthcare system.

## Supplementary Information

Below is the link to the electronic supplementary material.

Supplementary Material 1

## Data Availability

The data that support the findings of this study are available from the Merative™ MarketScan^®^ Research Databases but restrictions apply to the availability of these data, which were used under license for the current study, and so are not publicly available. Data are, however, available from the authors upon reasonable request from the corresponding author with permission from Merative^®^.

## Abbreviations

ACIP: Advisory committee on immunization practices
ACP: All-cause pneumonia
AOM: Acute otitis media
CDC: Centers for disease control and prevention
CMC: Chronic medical conditions
CPI: Consumer price index
ER: Emergency room
HCRU: Healthcare resource utilization
IC: Immunocompromising conditions
IP: Inpatient
IPD: Invasive pneumococcal disease
OME: Otitis media with effusion
OP: Outpatient
PCV: Pneumococcal conjugate vaccines
PD: Pneumococcal disease
PP: Pneumococcal pneumonia
PY: Person-years
US: United States
